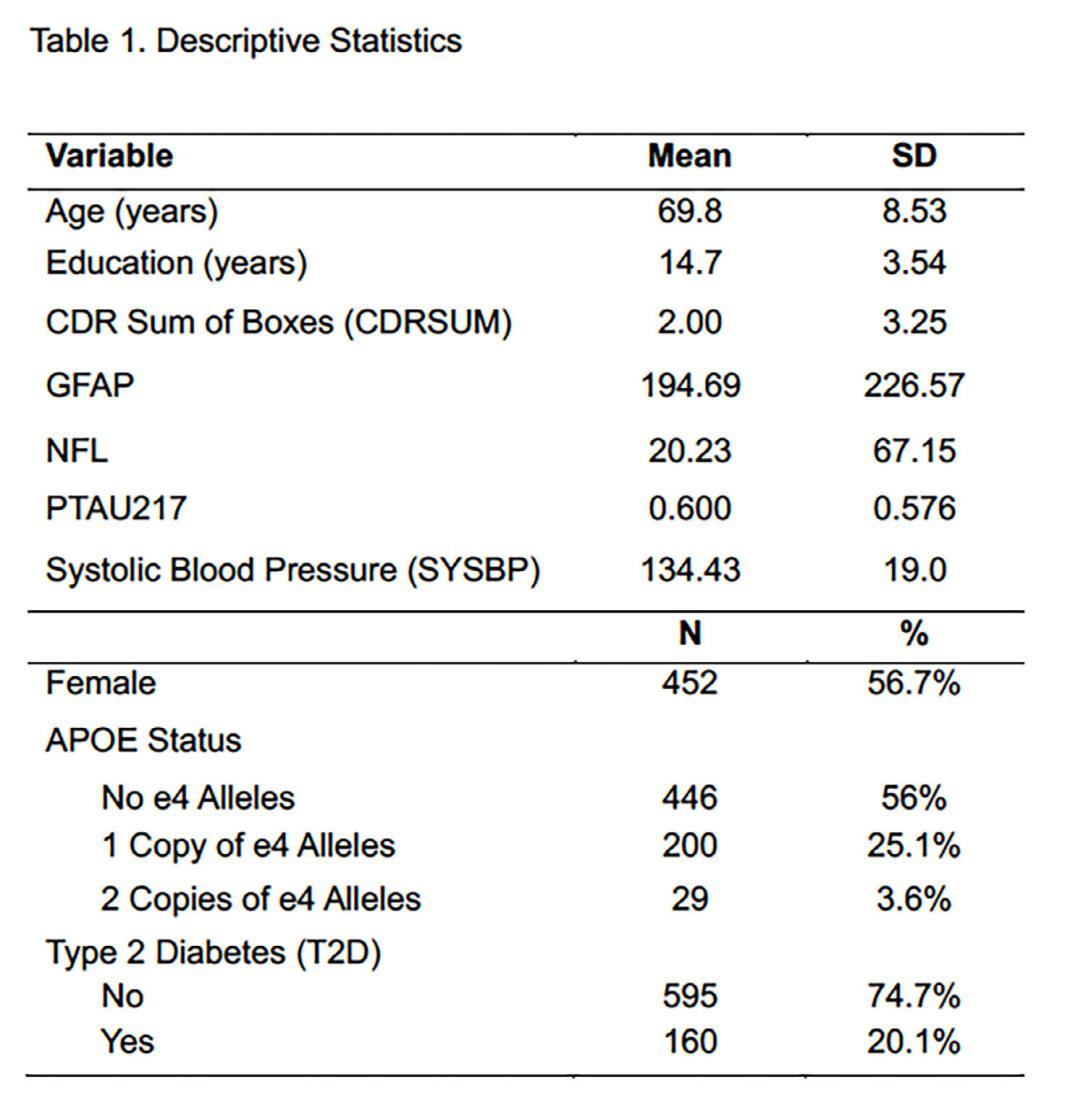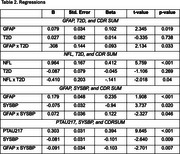# Systolic Blood Pressure and Type 2 Diabetes Modify the Relationship Between Alzheimer's Disease Biomarkers and Cognitive/Functional Impairment

**DOI:** 10.1002/alz70856_105329

**Published:** 2026-01-07

**Authors:** Tamare V Adrien, Krithika Sivaramakrishnan, Louisina Morancy, Ambar Perez‐Lao, Franchesca Arias, Breton M. Asken, Idaly Velez‐Uribe, Monica Rosselli, Melissa J. Armstrong, Rosie Elena Curiel, David A. Loewenstein, Ranjan Duara, Glenn E. Smith, Shellie‐Anne Levy

**Affiliations:** ^1^ University Of Florida, Gainesville, FL, USA; ^2^ University of Florida, Gainesville, FL, USA; ^3^ 1Florida Alzheimer's Disease Research Center, Department of Clinical and Health Psychology, University of Florida, Gainesville, FL, USA; ^4^ 1Florida Alzheimer's Disease Research Center, Miami, FL, USA; ^5^ Florida Atlantic University, Davie, FL, USA; ^6^ Florida Alzheimer's Disease Research Center, Gainesville, FL, USA; ^7^ Florida Atlantic University, Boca Raton, FL, USA; ^8^ University of Miami School of Medicine, Miami, FL, USA; ^9^ Mount Sinai Medical Center, Miami Beach, FL, USA

## Abstract

**Background:**

Vascular risk factors contribute to Alzheimer's disease (AD) dementia, but their interaction with AD pathology remains unclear. This study examined systolic blood pressure (SYSBP) and Type 2 Diabetes (T2D) as moderators of the relationship between plasma biomarkers and cognitive and functional impairment in older adults.

**Method:**

Data from 479 participants (Table 1.) in the 1Florida Alzheimer's Disease Research Center were analyzed. SYSBP and T2D were assessed, and plasma biomarkers included GFAP, NFL, Ptau181, and Ptau217. Linear regressions and post‐hoc simple slopes analyses examined whether SYSBP and T2D moderated biomarker associations with impairment, measured by the Clinical Dementia Rating Scale‐Sum of Boxes, controlling for age, sex, education, and APOE status.

**Result:**

See table 2. In brief, a significant positive interaction was found between GFAP and SYSBP on impairment (*b* = 0.074, *p* = .046), with GFAP significantly predicting impairment at all SYSBP levels (low *b* = 0.109, average *b* = 0.180, high b =0.250, *p* <.001) and a stronger association as SYSBP increased. In contrast, a significant negative interaction was observed between PTau217 and SYSBP (b = −0.091, *p* = .007), where PTau217 was significantly associated with impairment at all SYSBP levels but with a weakening effect as SYSBP increased (low b = 0.391, average *b* = 0.302, high *b* = 0.213, *p* <.001).

For T2D, a significant positive interaction was found between GFAP and T2D (b = 0.308, *p* = .033), with GFAP predicting impairment in both groups but showing a stronger effect in T2D. A significant negative interaction was observed between NFL and T2D (b = ‐0.410, *p* = .044), where NFL predicted impairment in both groups, though the association was weaker in T2D.

**Conclusion:**

These findings highlight vascular health as a key modifier of neurodegeneration and inflammation related impairment. The stronger GFAP‐impairment relationship at higher SYSBP and in T2D suggests astrocyte activation may drive decline more in individuals with vascular or metabolic comorbidities. In contrast, the weaker effects of PTau217 at high SYSBP and NFL in T2D suggest alternative mechanisms such as hyperperfusion and glycemic dysregulation. Future research should incorporate longitudinal designs, neuroimaging, and refined vascular risk assessments to clarify these relationships.